# Physiological mechanism of improved tolerance of *Saccharomyces cerevisiae* to lignin-derived phenolic acids in lignocellulosic ethanol fermentation by short-term adaptation

**DOI:** 10.1186/s13068-019-1610-9

**Published:** 2019-11-14

**Authors:** Hanqi Gu, Yuyong Zhu, Yanfang Peng, Xiujun Liang, Xiaoguang Liu, Lingzhi Shao, Yanyan Xu, Zhaohe Xu, Ran Liu, Jie Li

**Affiliations:** 1 Department of Biology and Food Science, Hebei Normal University for Nationalities, Chengde, 067000 Hebei China; 2 State Key Laboratory of Bioreactor Engineering, East China University of Science and Technology, 130 Meilong Road, Shanghai, 200237 China; 3 Basic Medical Institute, Chengde Medical University, Chengde, 067000 Hebei China

**Keywords:** *Saccharomyces cerevisiae*, Tolerance to phenolic acids, Lignocellulosic ethanol, Short-term adaptation, Cytoplasmic membrane integrity, Cytoplasmic membrane invagination

## Abstract

**Background:**

Phenolic acids are lignin-derived fermentation inhibitors formed during many pretreatment processes of lignocellulosic biomass. In this study, vanillic, *p*-hydroxybenzoic, and syringic acids were selected as the model compounds of phenolic acids, and the effect of short-term adaptation strategies on the tolerance of *S. cerevisiae* to phenolic acids was investigated. The mechanism of phenolic acids tolerance in the adapted yeast strains was studied at the morphological and physiological levels.

**Results:**

The multiple phenolic acids exerted the synergistic inhibitory effect on the yeast cell growth. In particular, a significant interaction between vanillic and hydroxybenzoic acids was found. The optimal short-term adaptation strategies could efficiently improve the growth and fermentation performance of the yeast strain not only in the synthetic media with phenolic acids, but also in the simultaneous saccharification and ethanol fermentation of corncob residue. Morphological analysis showed that phenolic acids caused the parental strain to generate many cytoplasmic membrane invaginations with crack at the top of these sites and some mitochondria gathered around. The adapted strain presented the thicker cell wall and membrane and smaller cell size than those of the parental strain. In particular, the cytoplasmic membrane generated many little protrusions with regular shape. The cytoplasmic membrane integrity was analyzed by testing the relative electrical conductivity, leakage of intracellular substance, and permeation of fluorescent probe. The results indicated that the short-term adaptation improved the membrane integrity of yeast cell.

**Conclusion:**

The inhibition mechanism of phenolic acid might be attributed to the combined effect of the cytoplasmic membrane damage and the intracellular acidification. The short-term adaptation strategy with varied stressors levels and adaptive processes accelerated the stress response of yeast cell structure to tolerate phenolic acids. This strategy will contribute to the development of robust microbials for biofuel production from lignocellulosic biomass.

## Background

**Fig. 1:**
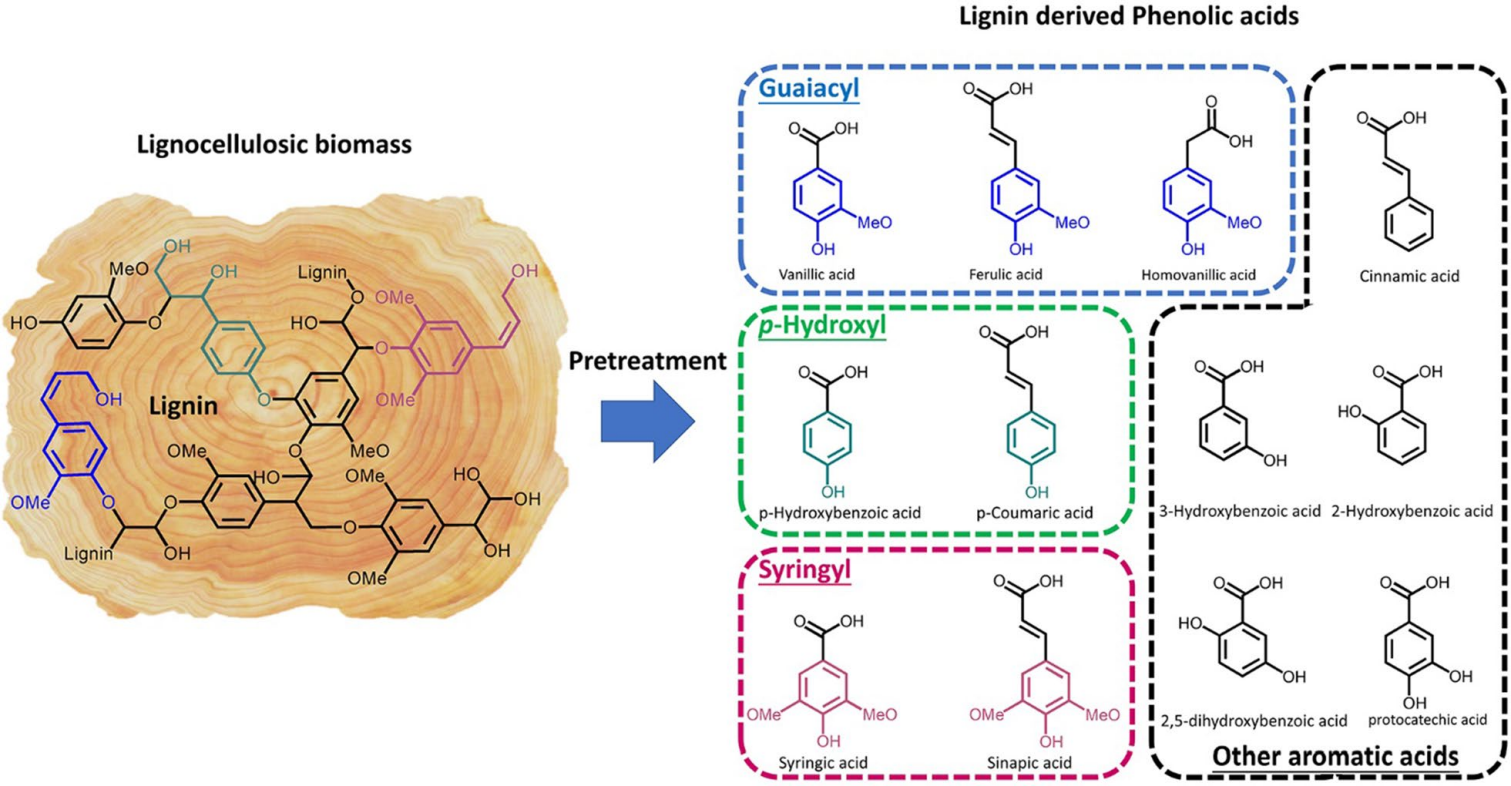
Chemical structure of phenolic acids found in lignocellulosic hydrolysates after pretreatment

The effect of lignocellulose-derived inhibitors on the fermenting microorganisms has become a challenge to the industrial development of biofuel production. Phenolic acids are the inhibitors with more kinds of compounds, higher concentration, and higher chemical stability than the phenolic aldehydes and alcohols in various lignocellulosic hydrolysate from common pretreatment methods (the dilute acid, steam explosion, alkali and alkaline wet oxidation) [1–4]. They are classified into groups of guaiacyl, *p*-hydroxyphenyl, syringyl, and other aromatic acids based on the structure of lignin monomers [5, 6] (Fig. 1). It has been found that phenolic acids were the main inhibitors that affected *S. cerevisiae* seriously during the simultaneous saccharification and ethanol fermentation of corn cob residue (SSF) [7, 8].

The inhibition mechanism of the phenolic acids remains not fully understood due to their complicated chemical structures [9, 10], although it was hypothesized that phenolic acids may damage the electrochemical gradient of the mitochondrial membranes [11, 12]. Many studies were conducted to compare the inhibitory effect of different phenolic acids [9, 13–15], but only a few of them studied the mechanism. Campos and colleagues reported the effects of phenolic acids on cell membrane permeability of lactic acid bacteria in wine [16]. Our previous study observed that ferulic acid could cause disaggregation of cytoplasmic membrane of *S. cerevisiae* [8]. Hence, further research on the mechanism of phenolic acids inhibition is needed for identifying their specific cellular target and further providing efficient solutions.

Short-term adaptation of yeast is an efficient strategy for improving strain tolerance towards lignocellulosic inhibitor by pre-exposed strain to the inhibitors present in the subsequent fermentation medium. This process incorporated to the seed cultivation process at low pH can efficiently simplify operation steps, decrease detoxification cost, and prevent bacterial contamination [17, 18]. In addition, it has been found that the concentration of lignocellulosic hydrolysate during adaptation process directly affects subsequent fermentation performance and cell biomass [19]. The adaptation strategies through pulse exposure to stress have been applied for improving the tolerance of *S. cerevisiae* to inhibitors and osmotic pressure [20, 21]. The short-term adaptation has been applied for improving the tolerance of *S. cerevisiae* to phenolic acids during the SSF of corn cob residue [7]. However, the effect of adaptation strategies on triggering and generating the tolerant phenotype to phenolic acids remains unclear. Moreover, the short-term adaptation to furfural could induce *Bacillus coagulans* to generate significant cell elongation, which related to the cell integrity and might be the reason for the tolerance to furfural [22].

In this study, the short-term adaptation strategies were used for improving the tolerance of *S. cerevisiae* to phenolic acids in the fermentation of lignocellulosic biomass, and the tolerance mechanism was revealed from the morphological and physiological traits of yeast cell. Specifically, the inhibitory effect of individual and multiple phenolic acids on the physiological properties of *S. cerevisiae* was investigated. The effect of adaptation strategies on the growth and fermentation performance of yeast strain was studied in the synthetic media with phenolic acids and in the SSF of corncob residue, respectively. The yeast cell structure was analyzed using scanning and transmission electron microscopy and flow cytometry. This study provided an efficient adaptation strategy for improving tolerance of yeast to phenolic acids and explained the reason at the cellular level.

## Results and discussion

### Inhibitory effect of individual phenolic acids on *S. cerevisiae*

Vanillic acid, *p*-hydroxybenzoic acid and syringic acid were selected as the model compounds of phenolic acids from the groups of guaiacyl, *p*-hydroxyphenyl and syringyl, respectively (Fig. 1) [23]. The growth and fermentation kinetic parameters of *S. cerevisiae* were investigated under the stress of individual phenolic acid (Table 1). With increasing concentration of phenolic acid, the cell growth presented the decrease in specific growth rate and biomass, and the increase in lag phase and growth inhibition. The minimum inhibitory concentrations (MIC) of vanillic and *p*-hydroxybenzoic acid were approximately 3.0 g/L and 5.0 g/L (equal to 17.8 mM and 36.2 mM). The growth inhibition caused by syringic acid at concentration more than 2.0 g/L could not be investigated due to its low solubility in water [7]. However, the MIC of syringic acid could be calculated as 3.8 g/L according to the previously reported method that extrapolation from the curve of specific growth rate against inhibitor concentration (Additional file 1: Figure S1) [14]. In addition, the fermentation performance indicated the obvious decrease in glucose uptake rate and ethanol productivity, while only slight inhibition on ethanol yield (Table 1).

**Table 1:** Effect of phenolic acids on the growth and fermentation performance of *S. cerevisiae*

Phenolic acids	Concentration (g/L)	*μ*_max_^b^ (h^−1^)	GI^b^ (%)	Lag phase (h)	*Y*_x/s_^b^ (g/g)	*Q*_glucose_ (g/L/h)	*Q*_EtOH_ (g/L/h)	*Y*_EtOH_ (%)
Vanillic acid	0	0.38	0	3	0.24	3.03	1.37	84.34
	0.33	0.35	7.78	3	0.21	3.07	1.25	81.17
	0.75	0.36	6.64	3	0.19	2.68	1.13	80.80
	1.5	0.33	13.99	3	0.16	2.47	0.88	84.65
	2.25	0.18	53.60	9	0.11	1.15	0.51	80.55
	3.0	0.00	99.55	15	0.03	0.78	0.24	61.14
*p*-hydroxybenzoic acid	0	0.37	0	3	0.25	3.00	1.27	82.79
	2.0	0.36	5.48	3	0.20	3.07	1.23	78.01
	2.5	0.33	12.20	3	0.18	3.00	1.25	80.36
	3.0	0.30	21.63	3	0.17	2.02	0.82	79.29
	4.0	0.20	45.88	9	0.14	1.17	0.50	82.62
	5.0	0	100	15	0.02	0.75	0.19	49.02
Syringic acid	0	0.38	0	3	0.26	2.87	1.26	85.96
	0.25	0.36	6.01	3	0.26	2.80	1.23	86.25
	0.5	0.36	5.05	3	0.23	2.62	1.17	84.62
	1.0	0.34	11.19	3	0.22	2.45	0.82	86.48
	1.5	0.30	20.98	3	0.20	2.40	0.61	83.11
	2.0	0.27	29.03	3	0.18	2.23	0.63	87.77
V–H–S^a^	0	0.37	0	3	0.26	2.82	1.27	86.06
	0.5–0.3–0.25	0.33	9.83	3	0.25	2.82	1.09	75.76
	1.0–0.6–0.5	0.23	38.77	3	0.20	1.92	1.05	71.06
	1.5–0.9–0.75	0.16	57.39	9	0.08	0.72	0.22	59.62
	2.0–1.2–1.0	0	100	15	0.03	0.10	0.08	21.36
	2.5–1.5–1.25	0	100	24	0.02	0	0	0

^a^V–H–S represented the cocktail of vanillic acid, *p*-hydroxybenzoic acid and syringic acid

^b^*μ*_max_ and *Y*_x/s_ represented the maximum specific growth rate and biomass yield during initial 15 h, respectively; GI is the growth inhibition rate

^c^*Y*_EtOH_ was the ratio of maximum ethanol concentration to the theoretical ethanol from the total glucose in the medium within 24 h of culture period

The result shows that the individual phenolic acid had an inhibitory effect on the growth and fermentation rate, but did not completely inhibit the final ethanol yield, which is consistent with previous studies about the inhibition of these phenolic acids on the yeast strains [2, 8, 15]. This is probably because the phenolic acid mainly caused the inhibition on the growth of yeast cells rather than directly inhibiting the central pathways for ethanol fermentation. A low level of the individual phenolic acid (vanillic acid, syringic acid and *p*-hydroxybenzoic acid were lower than 1.5, 2.0 and 3.0 g/L) did not affect the metabolism of glucose converted to ethanol (ethanol yield) in cell, but the rate of glucose uptake and ethanol productivity was limited by the cell growth rate, while high concentration of phenolic acid inhibited cell growth and ethanol fermentation simultaneously, leading to a decrease in ethanol yield. Moreover, the inhibition intensity order was vanillic acid > syringic acid >* p*-hydroxybenzoic acid. Although a similar result that phenolic acids inhibited the fermentation performance of *S. cerevisiae* was found, it did not study the inhibitory effect on the cell growth [24].

### Synergistic inhibition of multiple phenolic acids on *S. cerevisiae*

The effect of multiple phenolic acids on the growth and fermentation performance of yeast strain was investigated for analyzing their synergistic inhibition (Table 1). With increasing concentration of mixed phenolic acids, the maximum specific growth rate and biomass yield reduced from 0.37 h^−1^ and 0.26 g dry cell weight/g glucose to 0, respectively, and the lag phase was prolonged from 3 to 24 h. For the fermentation, the glucose uptake rate and ethanol productivity sharply decreased. In particular, the ethanol yield decreased from 86.06% to 0 with increasing concentration of mixed phenolic acids.

**Fig. 2:**
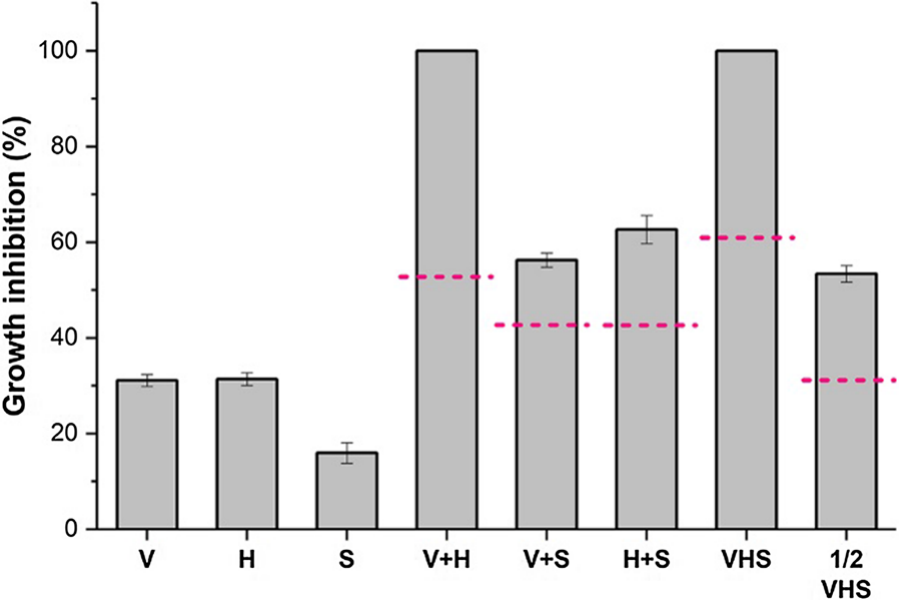
Synergistic inhibition of phenolic acids on growth of *S. cerevisiae*. V, H and S represented vanillic acid, *p*-hydroxybenzoic acid and syringic acid, which at concentrations of 2.1 g/L, 3.5 g/L and 1.2 g/L could cause approximately 30%, 30% and 15% of growth inhibition on the parental strain, respectively. V + H, V + S, H + S and VHS represented two or three kinds of these phenolic acids mixed at the above concentrations, and ½ means half of the concentration of the three phenolic acids. The dashed lines represented if growth inhibitions were theoretically additive. 52.7%, 42.3%, 60.2%, 30.1%

**Fig. 3:**
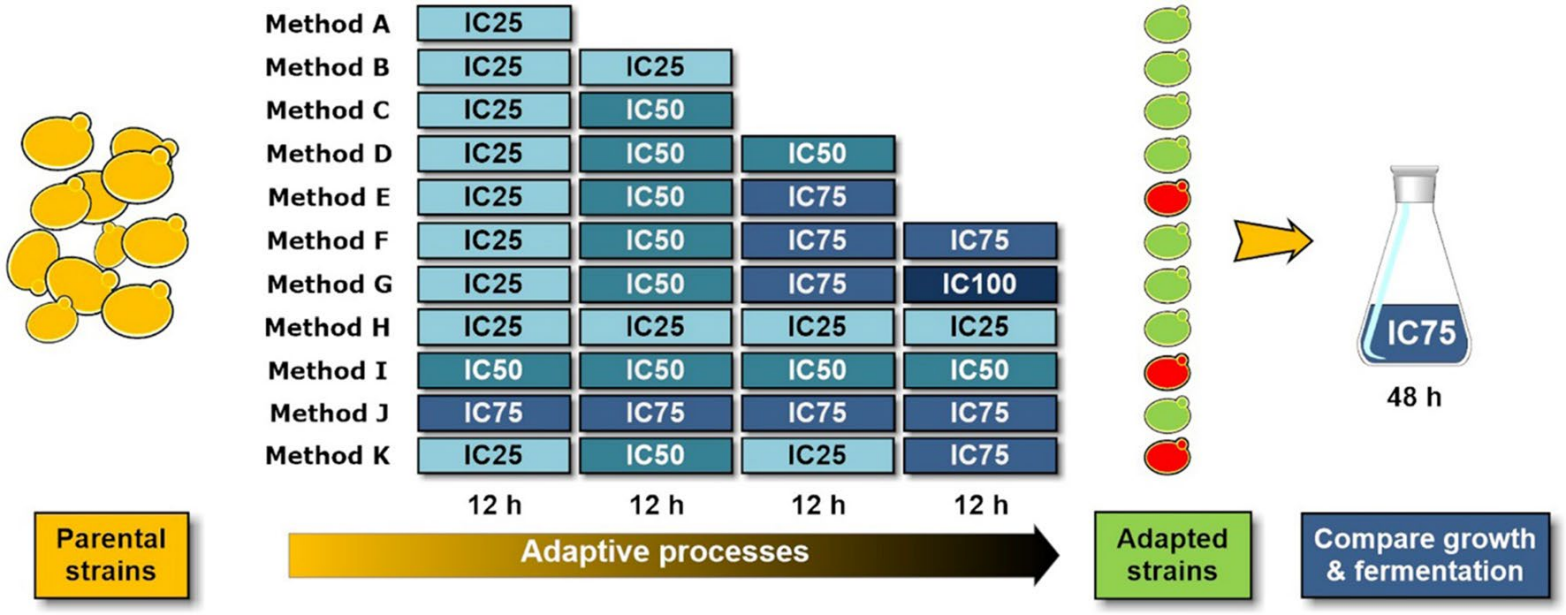
Scheme of the short-term adaptive processes of *S. cerevisiae* to the phenolic acids. The parental strain was cultured in a 100 mL flask containing 20 mL of sterilized synthetic medium at 10% (v/v) of inoculation, 30 °C, pH 6.0, for 18 h as the seeds culture. Then, yeast cells were sequentially transferring and culturing in synthetic medium contained phenolic acids mixture at the inhibitory concentrations from low to high. IC25, IC50, IC75 and IC100 represented the synthetic media contained phenolic acids at inhibitory concentrations which could cause 25%, 50%, 75% and 100% inhibition on growth rate of the parental strain. Each batch of culture was incubated at 10% (v/v) of inoculation in 100 mL flask contained 20 mL of phenolic acid media at desired inhibitory concentration, 30 °C with agitation at 150 rpm for 12 h. The adapted strains were incubated in the synthetic medium with glucose of 60 g/L and IC75 of phenolic acids mixture for comparing growth and fermentation performance, at 30 °C agitation rate of 150 rpm for 48 h

The synergistic inhibition of phenolic acids on the growth of *S. cerevisiae* was further investigated (Fig. 2). The individual vanillic, *p*-hydroxybenzoic and syringic acids at 2.1 g/L, 3.5 g/L and 1.2 g/L caused the growth inhibition rate of 31.1%, 31.4% and 15.9%, respectively. The dashed lines represented the theoretically additive inhibition caused by the mixed phenolic acids. For instance, the growth inhibition rate caused by the mixture of vanillic acid and *p*-hydroxybenzoic acid (V + H) should be (1 − 68.9% × 68.6%) = 52.7%, in which 68.9% and 68.6% were the growth rate of yeast cells under the stress of vanillic acid and hydroxybenzoic acid, respectively. However, the actual growth inhibition rate caused by V + H was 100%. The similar results show that the growth inhibition rates caused by the mixture of V + S, H + S, VHS and 1/2VHS were 56.3%, 62.7%, 100% and 53.4%, respectively. There was an obvious difference between the value of the actual growth inhibition and the theoretically additive inhibition (dashed lines in Fig. 3). These results show the multiple phenolic acids had synergistic inhibition on the cells growth and the ethanol fermentation, especially on the ethanol yield. These aligned with previous studies about the synergistic effect of phenolic acids on *Pichia stipites* and lactic acid bacteria [15, 25]. Moreover, the ANOVA result shows an interaction between vanillic and *p*-hydroxybenzoic acid is significant, which indicates that the inhibition of each other will be increased when they content in lignocellulosic hydrolysate simultaneously.

### Effect of short-term adaptation strategy on the tolerance to phenolic acids

**Fig. 4:**
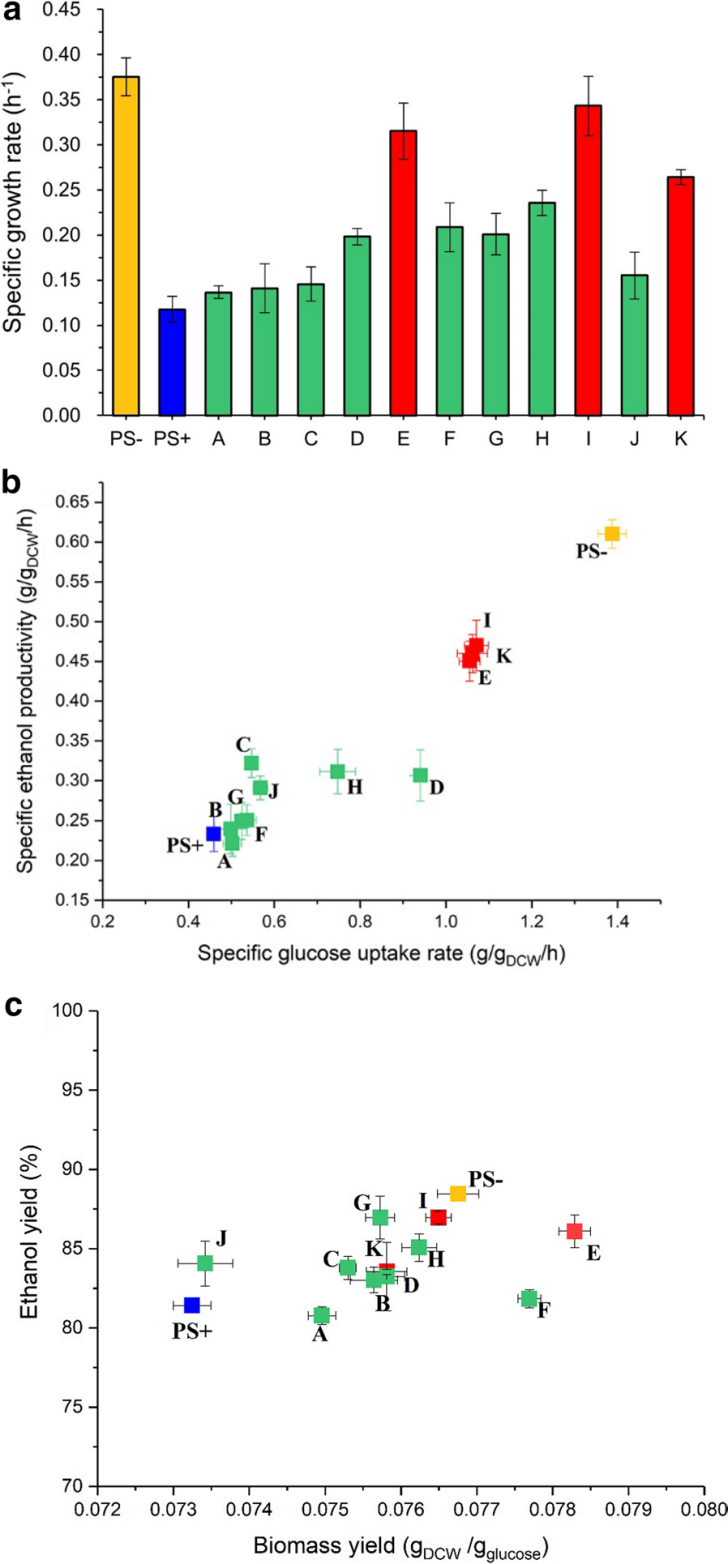
Physiological characterization of the phenolic acids adapted *S. cerevisiae* strains. The effect of phenolic acids mixture on **a** growth rate, **b** glucose consumption and ethanol production and **c** biomass yield based glucose and theoretical yield of ethanol. Fermentation condition: 10% (v/v) inoculation in the synthetic media with 60 g/L of glucose and IC75 of mixed phenolic acids at 30 °C, agitation rate of 150 rpm for 48 h. A–K represented the adapted strains from the adaptation methods A–K incubated under above condition; PS+ and PS− represented the parental strain incubated in synthetic medium with and without IC75 of mixed phenolic acids

The effect of short-term adaptation strategies, such as the stepwise adaptation to the increasing concentration (Method A–G), direct adaptation to constant concentration (Method H–J) and pulse adaptation to high concentration (Method K), on the tolerance of yeast strain to phenolic acids was investigated (Fig. 3). The specific growth rates of the adapted strains from methods E, I and K were 0.32, 0.34 and 0.26 h^−1^, respectively, which were 2–3 times higher than that of the parental strain under phenolic acid stress (Fig. 4a). The specific glucose uptake rate and ethanol productivity of the adapted strains from methods E, I and K were no obvious differences, which were about 2.33 and 1.92 times higher than those of the parental strain under the same condition, respectively (Fig. 4b). In addition, the ethanol yield and biomass yield of the adapted strains on average about 83.7% and 0.076 g dry cell weight/g glucose were slightly higher than those of the parental strain (Fig. 4c). This is probably because the parental strain could get the maximum yield of ethanol and biomass after the long lag phase during the 48 h’ fermentation.

**Fig. 5:**
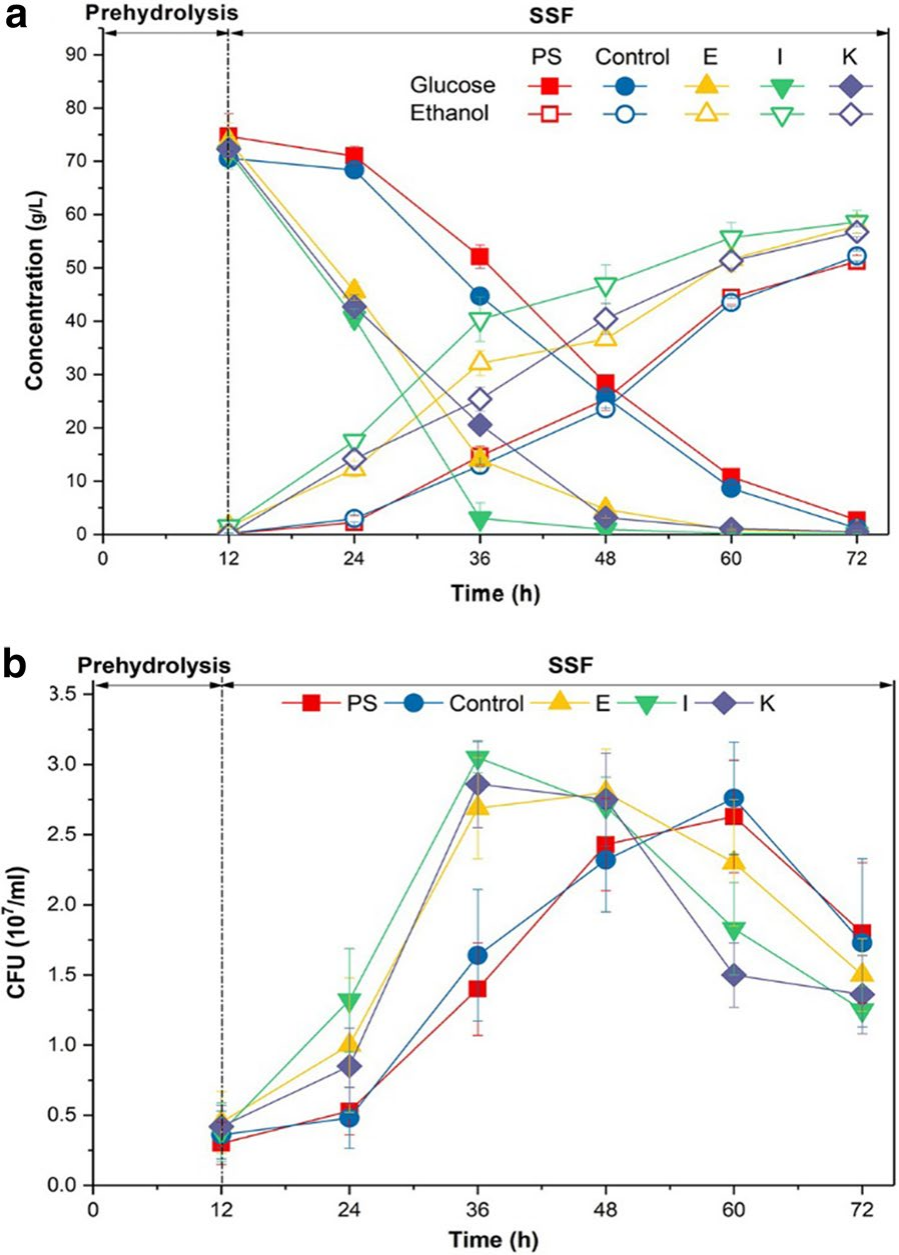
Adaptive processes on phenolic acids tolerance of *S. cerevisiae* during SSF of CCR. **a** Fermentation performance; **b** yeast cell growth. The colony-forming units (CFU) of the strains on petri dishes were counted for evaluation of cell growth viability. *PS* represents the parental strain cells; *Control* was the control group of adapted strain, which obtained from sequentially transferring and culturing parental strain cells in synthetic medium without phenolic acid with the same process as Method I; E, I and K were adapted strains from Method E, I and K, respectively. The prehydrolysis was performed at 50 °C and pH 4.8 for 12 h, then SSF was performed at 37 °C at pH 5.5. The cell growth was represented by the colony-forming units (CFU) of the strains on petri dishes

The corncob residue as a real lignocellulosic biomass contained a considerable amount of phenolic acids(16.8 mg/g dry mater), mainly including *p*-hydroxybenzoic acid, *p*-coumaric acid, ferulic acid, guaiacol and 2-furoic acid [7]. The adapted strains from methods E, I and K were selected to carry out the SSF of corncob residue for further analyzing their tolerance to the complex phenolic acids. The ethanol productivity of adapted strains from methods E, I and K were 1.10, 1.63 and 1.35 g/L/h during the initial 12 h, which were much higher than that of the parental strain and control group (continuously transferring and culturing yeast parental strain cells in synthetic medium with the same process as Method I) (Fig. 5a). In addition, the cell growth of the adapted strains from methods E, I and K reached the highest colony-forming units (CFU) of 2.69, 3.05 and 2.86 × 10^7^ in the initial 24 h of fermentation, while the parental strain and control group obtained the highest CFU of 2.63 and 2.75 × 10^7^ for 48 h. Then, the cell viability of the parental and adapted strains began to decrease (Fig. 5b), which might be caused by the synergistic inhibition of ethanol and phenolic acids. Moreover the ethanol produced by yeast strain might lead to more phenolic acids released from the lignocellulosic feedstock during the SSF progresses [26].

The results indicate that the relationship between ethanol production and cell growth rate is closer than to cell mass under the phenolic acid stress. The cell growth rate could be applied as one of the most important kinetic parameters for determining the tolerance of yeast strain to phenolic acids. methods E, Is and K represented the three types of short-term adaptation strategies could effectively improve the tolerance of *S. cerevisiae* to phenolic acids. This is similar to the previous studies that the stepwise and pulse adaptation strategies improved the tolerance of *S. cerevisiae* to inhibitors and high-osmolarity [20, 21]. The stress response of microbials probably depends on the activation of defense and repair mechanisms for a specific stress [27, 28]. The adaptation methods E, I and K under the phenolic acids’ stress at range from IC50 to IC75 might trigger the response of tolerant phenotype, while above this concentration probably caused the irreparable cell damage. Therefore, the adaptive process and stressor level might play the key role on the short-term adaptation for improving tolerance to the inhibitors. In addition, the growth and fermentation performance of the adapted strains in the SSF process indicated that the short-term adaptation could accelerate stress response of yeast cells to the phenolic acids during the initial fermentation. However, the tolerant phenotype of the adapted strains could not be stably trasferred to the offspring after removing the phenolic acids stress (data not shown). This phenomenon may be explained by the mechanism of epigenetic transcriptional memory, which can be transferred to 6–7 generations through cell division [29, 30]. The yeast cells that have been exposed to periodic changes of phenolic acids probably acquired the “memory” of previous experiences. When exposed the adapted strain to the same stress of phenolic acids, the related gene expression and physiological changes will be triggered to fast stress response. The specific mechanism of transcription and expression will be further studied. Moreover, the result of short-term adaptation performed in the shake flask under uncontrolled conditions of aeration and pH provides only an indication about the possible behaviors of yeast strain in a controlled fermentor. The previous studies reported some other strains presented different physiological behaviors under the culture conditions with and without control [31, 32]. Therefore, the short-term adaptation of yeast to phenolic acids will be performed in the fermentor for the further study to provide a more accurate prediction about the behavior of their strain when this strategy is used in the real industrial production of bioethanol.

### Morphology analysis of adapted strain

**Fig. 6:**
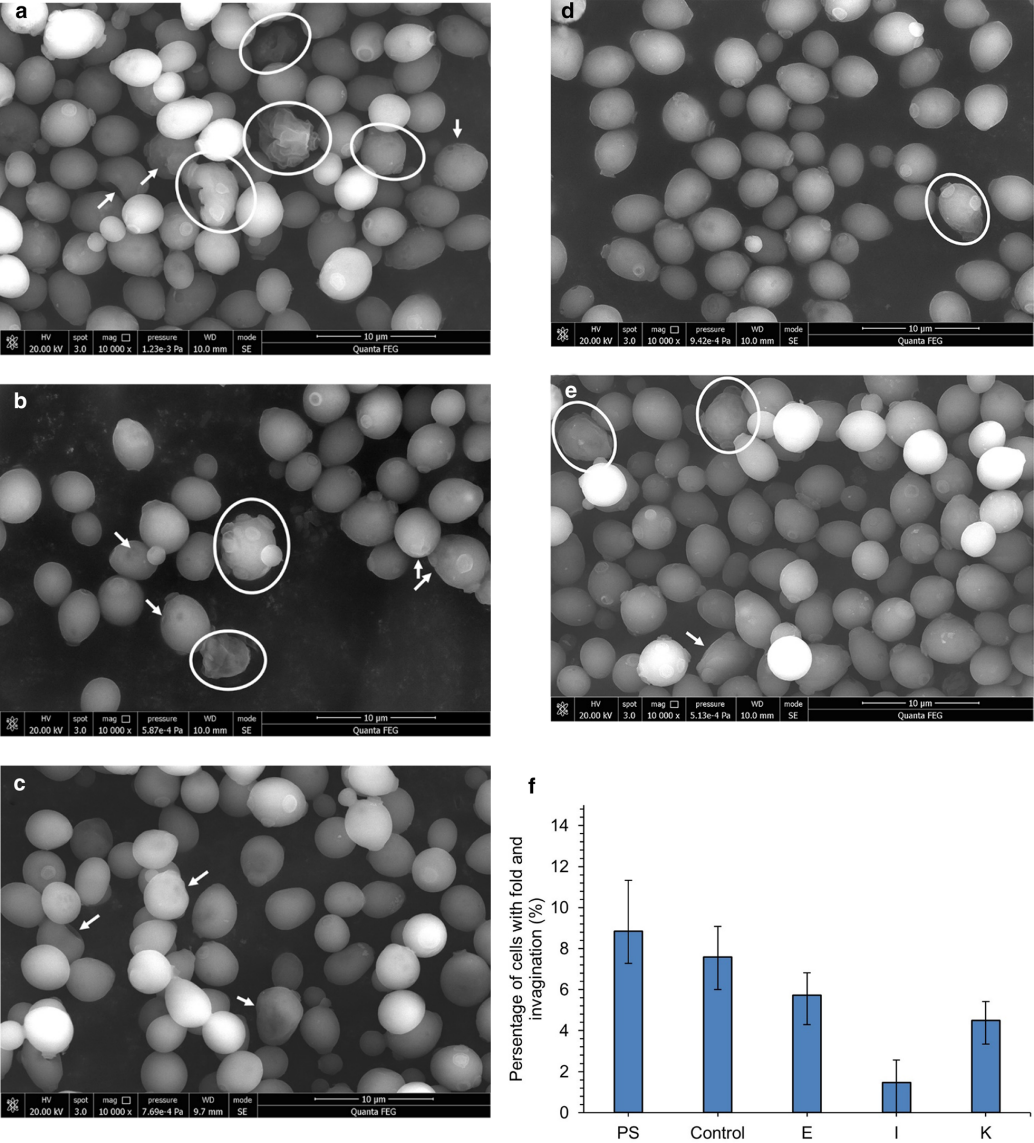
SEM of *S. cerevisiae* after treatment of phenolic acids. **a** The parental strain cells, **b** the control group of adapted strain, which obtained from sequentially transferring and culturing yeast parental strain cells in synthetic medium without phenolic acid at the same condition with Method I; **c**–**e** were adapted strains from Method E, I and K, respectively. All above strain cells were inoculated in the medium with phenolic acids mixture at concentration of IC75 at 30 °C with agitation at 150 rpm for 9 h. Circle marks the folds on the cell surface; Arrow marks the invagination of cell. **f** Percentage of cells with fold and invagination. Each sample was observed in three different area (35–90 cells), the percentage of cells with fold and invagination was calculated by the mount of cells with fold and invagination divided total cells in the observed area

The cell morphology and internal structure of the adapted and parental under the mixed phenolic acids’ stress were observed using the scanning electron microscopy (SEM) and transmission electron microscopy (TEM) to analyze the phenolic acids’ tolerance. The SEM images showed that most cells of the parental and adapted strains were of smooth ellipsoidal shape (Fig. 6). Certain cells of the parental strain and the control group of adaptive process showed obvious invaginations and folds on the surface (marked by arrows and circles), which were more than those of the adapted strains from methods E, I and K (Fig. 6f).

**Fig. 7:**
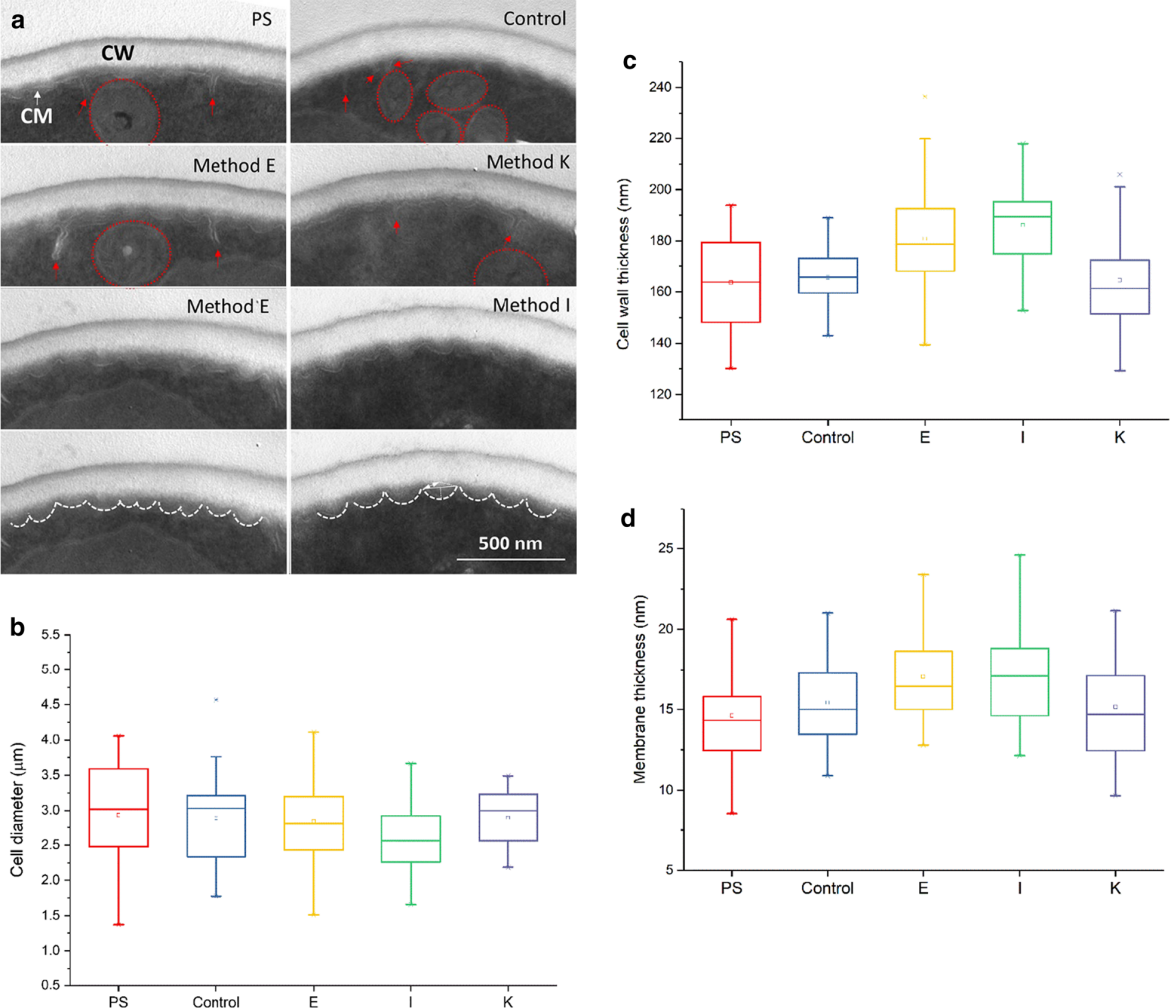
TEM imagines of *S. cerevisiae* after treatment of phenolic acids. **a** the magnified specific parts of yeast strain cells; **b**–**d** quantification of cell diameter, thickness of cell wall and membrane (measurement of over 50 cells), respectively. *PS* represents the parental strain cells; *control* was the control group of adapted strain, which obtained from sequentially transferring and culturing parental strain cells in synthetic medium without phenolic acid at the same condition with Method I; E, I and K were adapted strains from Method E, I and K, respectively. All above strain cells were inoculated in the medium with phenolic acids mixture at concentration of IC75, 30 °C, 150 rpm for 9 h. *CW* cell wall, *CM* cytoplasmic membrane, red circle marks mitochondria; red arrow marks cytoplasmic membrane invagination

The TEM images showed apparent changes in the internal cellular structure (Fig. 7). The cells of the parental and control group strains showed many cytoplasmic membrane invaginations and the small fragments of vacuole in lobular shape, while the cells of adapted strains (from Method E, I and K) presented the normal cell morphology with little invaginations (Additional file 1: Figure S2). It was worth noting that the obvious disruption or crack was found at the top of cytoplasmic membrane invaginations (marked by the arrows) and the mitochondria (white circle dash line) surround this area (Fig. 7a). In addition, the cells from adaptive methods E and I showed the thicker cell wall and membrane (Fig. 7c, d) and the little protrusions with regular shape on cytoplasmic membrane (Fig. 7a), while the mean diameter of cells was slightly smaller than that of the other strains (Fig. 7b).

The results indicate that the inhibitory effect of phenolic acids on *S. cerevisiae* probably was caused by their aromatic group and carboxylic acid. The hydrophobic group might insert the phospholipid molecular layer, leading to loss of membrane integrity [8, 33, 34]. Carboxylic acid group of phenolic acid entered the cytoplasmic membrane and dissociated to form hydrogen ion (H+), which caused the intracellular pH disorder [35]. The mitochondria-surrounded cytoplasmic membrane invaginations indicated that cells needed more energy to keep the constant intracellular pH by ATPase pumping out excess protons [36, 37]. In addition, combined with the results that the adapted strains grew faster than the parental strain in the initial 12 h of SSF (Fig. 5b), the adapted strains might trigger stress response faster than the parental strain. The changes in the structure of cell wall and membrane possibly provide a strong cell barrier to resist the phenolic acids [38–40]. Yeast strain could decrease cell volume and increase cell wall thickness by structural realignment of cell wall polysaccharide [41] and improve membrane integrity by cytoplasmic membrane proliferation [42]. Therefore, the short-term adaptation possibly accelerates the stress response of yeast cell structure for improving the tolerance to the phenolic acids.

### Cytoplasmic membrane integrity

The cytoplasmic membrane permeability and integrity were evaluated by testing the relative electrical conductivity, leakage of intracellular substance and cells stained by propidium iodide (PI) fluorescent dye.

**Fig. 8:**
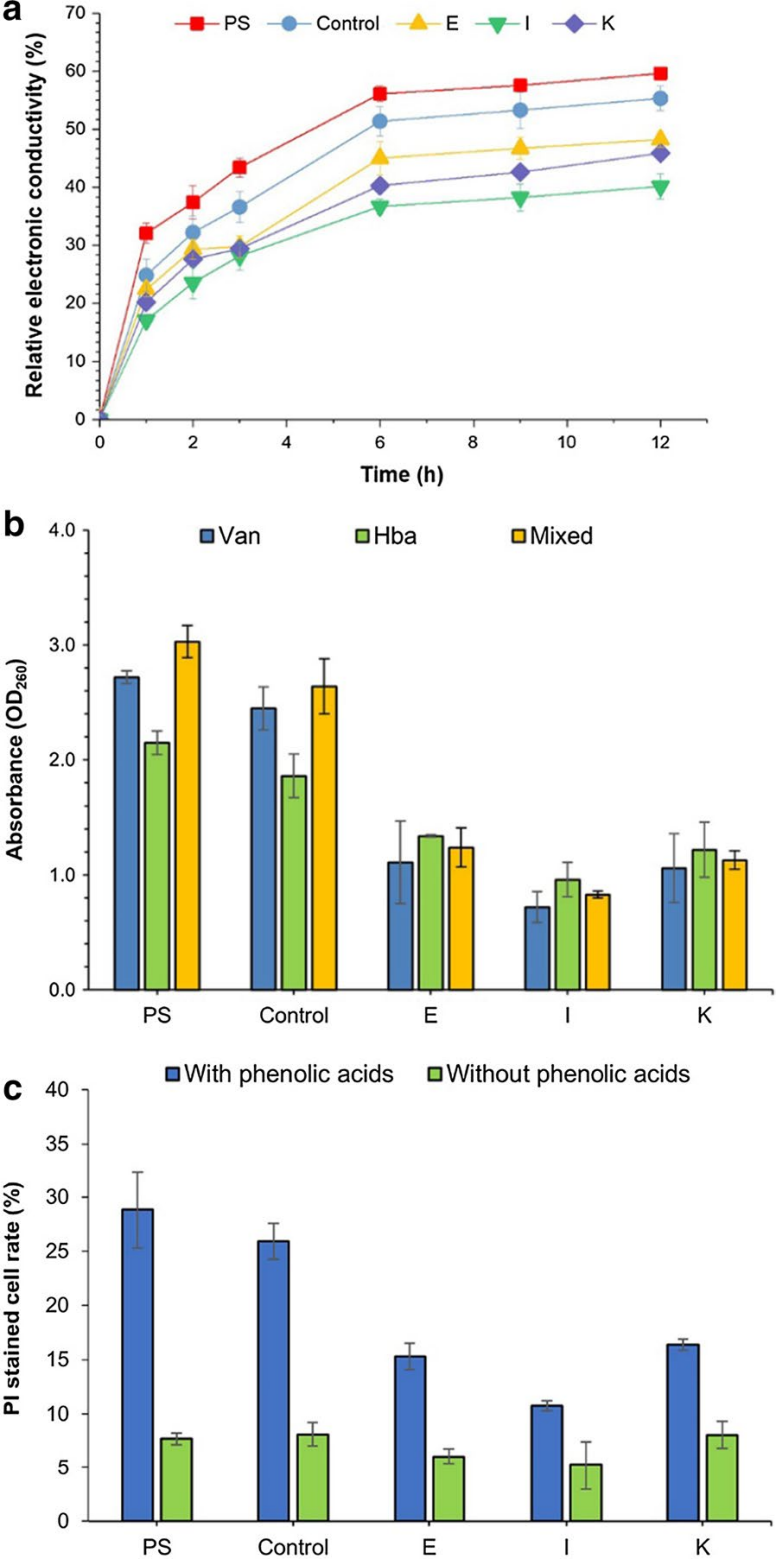
Cytoplasmic membrane integrity of yeast strains under phenolic acids stress. **a** relative electric conductivity; **b** leakage of intracellular 260 nm-absorbing substances; **c** PI stained cells rate; *PS* represents the parental strain cells; *Control* was the control group of adapted strain, which obtained from sequentially transferring and culturing yeast parental strain cells in synthetic medium without phenolic acid at the same condition with Method I; E, I and K were adapted strains from Method E, I and K, respectively. All above yeast strains were treated in PBS (0.1 M, pH = 7.4) with phenolic acids mixture at inhibitory concentration of IC75 at 30 °C for desired time before above detections. Van, Hba and Mix represented vanillic acid, *p*-hydroxybenzoic acid and phenolic acids mixture, respectively

The electrical conductivities of all strains increased rapidly during the initial 3 h, and then the increase tended to slow down. The relative electrical conductivities of the adapted strains from methods E, I, K were approximately 40% until 12 h, which were lower than that of the parental strain (59.6%) and control group strain (55.3%) (Fig. 8a). In addition, the OD260 (optical density at 260 nm) of the intracellular substances leaked from the adapted strains were approximately 0.72–1.34, which were lower than those of the parental strain and control group strain (Fig. 8b). Moreover, PI is an indicator of cytoplasmic membrane integrity for it can penetrate the damaged cytoplasmic membrane and form a fluorescent complex by banding with DNA or RNA. Under the phenolic acids stress, the PI stained cells of the parental strain and control group increased obviously to 28.5% and 26%, while those of the adapted strains from methods E, I and K were 10.3%, 15.4% and 16.2%, respectively (Fig. 8c).

The result suggests that the phenolic acids could affect the cytoplasmic membrane integrity of the yeast strains and cause cytoplasmic membrane to lose the selective permeability barrier function, which is agreeable with the previous study about effect of ferulic acid on *S. cerevisiae* and *Z. mobilis* [8]. Moreover, the adapted strains presented the greater membrane integrity than the parental strains under the phenolic acids stress. Similar result was reported in the adapted *S. cerevisiae* with high tolerance to a mixture of acetic acid, furfural and phenol [43]. Therefore, the improvement of cytoplasmic membrane integrity may contribute to the tolerance of the adapted strain to the phenolic acids.

## Conclusion

Phenolic acids presented a significantly synergistic inhibition on the cell growth and ethanol fermentation performance. The toxic mechanism of the phenolic acid to *S. cerevisiae* might be the combined effect of the cytoplasmic membrane damage caused by its hydrophobic aromatic ring and the intracellular acidification induced by dissociation of its carboxylic acid group. In addition, the phenolic acids level and the adaptive process of the short-term adaptation method played an important role in accelerating the stress response in yeast cell structure. The changes in cell morphology could maintain the cytoplasmic membrane integrity for improving the tolerance to the phenolic acids. The short-term adaptation strategies efficiently improve the ethanol productivity and cell growth viability during the SSF of corncob residue with rich phenolic acids.

## Methods

### Strain and chemicals

*Saccharomyces cerevisiae* used in this study was a commercial active dry yeast (product No. 80000012, Angel Yeast Co., Ltd., Yichang, China) with fast growth and fermentation rate [43, 44]. The strain was cultured in synthetic medium including 20.0 g/L of glucose, 2.0 g/L of KH_2_PO_4_, 1.0 g/L of (NH_4_)_2_SO_4_, 1.0 g/L of MgSO_4_·7H_2_O and 10 g/L of yeast extract. The culture solution was aliquoted into 2.0 mL cryopreservation vials with 30% (w/w) glycerol and stored at − 80 °C freezer.

Yeast extract was purchased from Oxoid (Hampshire, England). Vanillic acid, *p*-hydroxybenzoic acid, syringic acid, propidium iodide were analytical pure and purchased from Sigma-Aldrich Trading Co. (Shanghai, China). All other chemicals were analytically pure and obtained from Tianjin Kemiou Chemical Reagent Co. Ltd (Tianjin, China).

### Analysis of inhibition of phenolic acids on *S. cerevisiae*

The inhibition of phenolic acids on the growth and fermentation of *S. cerevisiae* was investigated by culturing the strain in the inhibitor media containing phenolic acids. The stock solution of single or multiple phenolic acids including vanillic acid, *p*-hydroxybenzoic acid and syringic acid was added into the sterilized synthetic medium at desired concentration for inhibition studies. Dimethyl sulfoxide was used as cosolvent in the inhibitor medium with concentration below 0.7% (v/v) and without toxic effect on *S. cerevisiae* at this concentration [7, 14]. The strain was incubated in 100 mL flask that contained 20 mL of inhibitor media at desired concentration (Table 1) with 10% (v/v) of inoculation at 30 °C, 150 rpm for 24 h. For the study on synergistic inhibition of phenolic acids, a three-factor, two-level factorial design was preformed using the growth inhibition rate as response factor with concentrations of vanillic acid, *p*-hydroxybenzoic acid and syringic acid at 0 and 2.1 g/L, 0 and 3.5 g/L, 0 and 1.2 g/L, respectively. Samples were taken from shake flasks at desired time and centrifugated at 4427×*g* for 5 min. Optical density of strain cell was analyzed by spectrophotometry at 600 nm. A linear relationship between optical density and dry cell weight was 0.51 g/L dry cell weight corresponding to the OD_600_ of 1.0. The supernatant was stored at − 20 °C for the fermentation metabolites’ analysis by HPLC. The synergistic inhibition on growth inhibition rate was statistically analyzed by Design-Expert version 8.0.6 from Stat-Ease, Inc. (Minneapolis, MN).

### Determination of the growth and fermentation performance

The effect of phenolic acids on cell growth of *S. cerevisiae* was evaluated by specific growth rate (*μ*_max_), growth inhibition rate (GI) and biomass yield (*Y*_x/s_).

The specific growth rate was calculated by the following formula:

1 $$\mu_{\text{max} } = \frac{{\ln \left( {x_{t} /x_{c} } \right)}}{t},$$

where *μ*_max_ is the maximum specific growth rate during the log phase (h^−1^), *x*_*t*_ is the dry cell weight at the time *t* (g/L), *x*_*c*_ is the initial dry cell weight concentration (g/L), and *t* is the time interval between *x*_*c*_ and *x*_*t*_ (h) [45].

The growth inhibition rate was calculated by the following formula:

2 $${\text{GI}} = \left( {1 - \frac{{\mu_{i} }}{{\mu_{\text{sm}} }}} \right) \times 100\% ,$$

where GI is the growth inhibition rate of strain cultured in the inhibitor media of phenolic acids, *μ*_*i*_ and *μ*_sm_ are the maximum specific growth rate of strain cultured in the inhibitor media of phenolic acids and synthetic medium, respectively (h^−1^). Especially, minimum inhibitory concentrations (MIC) were defined as the lowest concentration of phenolic acids which could cause complete inhibition on yeast cell growth within a 24 h’ cultivation [46].

The *Y*_x/s_ was obtained as the weight of maximum biomass divided by the total consumed glucose.

The effect of phenolic acids on fermentation performance of *S. cerevisiae* was evaluated by specific glucose uptake rate (*q*_glucose_), specific ethanol productivity (*q*_EtOH_) and ethanol yield (*Y*_EtOH_). The *q*_glucose_ and *q*_EtOH_ were the ratio of the maximum glucose uptake rate (*Q*_glucose_) and ethanol productivity (*Q*_EtOH_) to the maximum dry cell weight, respectively. The *Y*_EtOH_ was the ratio of actual ethanol concentration to the theoretical ethanol from the total glucose in the medium.

Average values of biological replicates were used as the final yield for each culture condition.

### Short-term adaptation to phenolic acids

The strain was cultured in a 100-mL flask containing 20 mL of sterilized synthetic medium at 10% (v/v) of inoculation, 30 °C, pH 6.0, for 18 h as the seeds culture.

The various short-term adaptive processes of *S. cerevisiae* to the phenolic acids mixture were performed according to the previous study [7]. Briefly, the adaptive process was carried out by sequentially transferring and culturing yeast cells in synthetic medium containing phenolic acids mixture at the inhibitory concentrations from IC25 to IC75 (Table 2). During the adaptive processes each batch of culture was incubated at 10% (v/v) of inoculation in 100 mL flask contained 20 mL of phenolic acid media at desired inhibitory concentration with pH range of 4.8–5.2, 30 °C with agitation at 150 rpm for 12 h. The adaptive processes were named from A to K according to the increase in culture batches and inhibitory concentrations during the adaptation. The growth and fermentation performance of the adapted strains were investigated and compared by incubated in the synthetic medium contained phenolic acids at concentration of IC75 (Fig. 3).

**Table 2:** Concentrations of combined phenolic acids in synthetic media

	Concentrations of phenolic acids in adaptation media (g/L)		
	Vanillic acid	*p*-hydroxybenzoic acid	Syringic acid
IC25^a^	1.16	0.70	0.58
IC50	1.40	0.84	0.70
IC75	1.60	0.97	0.80
IC100	1.90	1.14	0.95

^a^IC25, IC50, IC75 and IC100 represented the synthetic media contained phenolic acids at inhibitory concentrations which could cause 25%, 50%, 75% and 100% inhibition on growth rate of the parental strain. The concentration of vanillic acid, p-hydroxybenzoic acid and syringic acid in the original mixture of phenolic acids was 2.5, 1.5 and 1.25 g/L and gradually diluted to different concentration level with the constant ratio shown as Table 1. A fitting curve of growth inhibition rate (GI) and dilution times was established based on the data of Table 1. The concentration of phenolic acids mixture led to 25%, 50% or 75% of growth inhibition was obtained by putting the growth inhibition rate of 25%, 50% and 75% into the equation of the fitting curve, respectively, and calculated the corresponding dilution times

For testing genetic stability of the improved tolerance to phenolic acids, the yeast cells of adapted strain were transferred and cultured in the synthetic medium without phenolic acids for five batches (each batch of 12 h), and then were collected and added into the medium with mixed phenolic acids with the commercially available strain as control. Culture conditions were same as above mentioned.

### Simultaneous saccharification and ethanol fermentation (SSF)

The SSF of corncob residue was performed in a 5-L bioreactor equipped with a helical impeller according to the previous study described by Zhang et al. [47]. The corncob residue contained 66.3% of water and 33.7% of total solids (the amount of solids remaining after heating the sample at 105 °C to constant weight [48]). The total solids of corncob residue contained 56.5% of glucan and 2.6% of xylan. Prehydrolysis of corncob residue was carried out with solids loading of 25% (g/g, weight of total solids of corncob residue/total weight of the SSF system. for example, 741.8 g corncob residue contained 250 g of total solids in the total 1000 g of the SSF system), cellulase dosage of 15 FPU/g total solids of corncob residue at 50 °C and pH 4.8 for 12 h. Then, SSF was performed at pH 5.5, 37 °C for 60 h by added the yeast strains into bioreactor with the initial OD_600_ of 7.5–8.1 and 10% of inoculation. About 1.5 mL of fermentation slurry (mixture of solid and liquid) was taken from the sampling port of bioreactor by a small wine dipper ladle spoon with long handle under the flam condition at desired time for the analysis of HPLC and cell growth. All the data are the mean values with standard deviations of the duplicate experiments.

The cell growth in the SSF process was measured by counting colony-forming units (CFU) [8]. 100 μL of diluted fermentation slurry (10^5^) was spread on the solid synthetic medium and incubated at 30 °C for 48 h, and then the single colony on each dish was counted. An average value of triplicate samples represented for the cell growth performance.

### HPLC analysis

Glucose, ethanol, acetic acid and glycerol were analyzed using LC-20AD HPLC (Shimadzu, Kyoto, Japan) equipped with RID-20A refractive index detector and Bio-Rad Aminex HPX-87H column at 65 °C. The mobile phase was 5 mM H_2_SO_4_ at 0.6 mL/min [45]. All samples were centrifuged at 11,167×*g* for 5 min and the supernatant filtered through a 0.22-mm filter before analysis.

### Electron microscopy observation

The cell morphology of *S. cerevisiae* was examined using the scanning electron microscopy (SEM) and the transmission electron microscopy (TEM). The yeast cells were collected after the incubation in the inhibitor medium of phenolic acidsmixture at inhibitory concentration of IC75 at 30 °C, 150 rpm for 9 h. The process of sample preparation was carried out according to the previous study [8]. Briefly, the yeast cells were fixed with 2% glutaraldehyde for 30 min and dehydrated by a graded ethanol series, air-dried, and sputtered coat with 1–2 nm gold. The treated cells were observed on a FEI Quanta 450FEG SEM (FEI, Hillsboro, USA) at the accelerating voltage of 20 kV.

The sample preparation for TEM analysis was similar to that for SEM analysis with some exception as follows: the yeast cells were post-fixed with 2% osmium tetraoxide (OsO4) for 1 h, then dehydrated, embedded, sectioned, and mounted on the copper grids, after that stored in a desiccator before microscopic examination. Subsequently, the ultrathin sections were imaged by a JEM 1200EX TEM (JEOL, Tokyo, Japan) at the accelerating voltage of 80 kV. The mean thicknesses of cell wall and cytoplasmic membrane (measured 20 cells, 30 different location of each cell) and cell diameter (measured more than 40 cells) were measured using Image-J.

### Cytoplasmic membrane integrity

The yeast cytoplasmic membrane permeability and integrity were evaluated according to the methods of analyzing the relative electrical conductivity, leakage of intracellular nucleotides and the propidium iodide (PI) stained cells described by with modification. The yeast strains were treated in PBS (0.1 M, pH = 7.4) with phenolic acids’ mixture at inhibitory concentration of IC75 at 30 °C for desired time before above detections. The PBS (pH 7.4, 0.1 M) was selected to remove the effect of pH, osmotic pressure and other salt ions in the medium on the cytoplasmic membrane [49–51].

The relative electrical conductivity was analyzed as following: yeast cells (approximately 1 × 10^7^ cells/mL) were treated with the mixed phenolic acids for 12 h. The samples were taken at the desired time and centrifuged at 4427×*g* for 5 min. The electrical conductivity of supernatant was measured using a DDS-307 conductometer (INESA Scientific Instrument Co., Ltd., Shanghai, China). The relative electrical conductivity was calculated according to the following equation:

3 $${\text{Relative}}\;{\text{electrical}}\;{\text{conductivity}} = \frac{{K_{1} - K_{0} }}{{K_{2} - K_{0}^{'} }} \times 100\%$$

where the electrical conductivity of sample supernatant was recorded as *K*_1_, and that of PBS with the mixed phenolic acids was recorded as *K*_0_. The electrical conductivity of the yeast cells incubated in the PBS and boiled for 5 min after taken at the desired time was recorded as *K*_2_, and that of PBS was recorded as *K*_0_′.

The leakage of intracellular nucleotides was analyzed as following: yeast cells (approximately 1 × 10^7^ cells/mL) were treated the phenolic acids mixture for 3 h. The release of nucleotides was measured at 260 nm by a 752-N spectrophotometer (INESA Analytical Instrument Co., Ltd, Shanghai, China), and the PBS contained phenolic acids mixture was used as a blank.

Flow cytometry analysis: after being treated with the phenolic acids mixture for 9 h, yeast cells (approximately 10^7^ cells/mL) were stained with PI (1 μg/mL) and incubated in the dark at 4 °C for 15 min. The stained cells were washed three times and measured using BD FACSCalibur™ flow cytometer with CellQuest Pro software (BD Biosciences, Franklin Lakes, NJ, USA). 20,000 cells were collected at the flow rate of fluidics at 35 μL/s. The excitation was at 488 nm, and PI emission was collected at 585/40 nm.

## Supplementary information

**Additional file 1: Figure S1.** Effect of phenolic acids on the specific growth rate. **Figure S2.** TEM imagines of *S. cerevisiae* after treatment of phenolic acids.

## Data Availability

The datasets used and/or analyzed during the current study are available from the corresponding author on reasonable request.

## Abbreviations

CFU: colony-forming units
GI: growth inhibition rate
HPLC: high-performance liquid chromatography
H: *p*-hydroxybenzoic acid
H + S: *p*-hydroxybenzoic acid and syringic acid
IC: inhibition concentration
*K*: electrical conductivity
MIC: minimum inhibitory concentrations
OD: optical density
PI: propidium iodide
*Q*_glucose_: glucose uptake rate
*Q*_EtOH_: ethanol productivity
*q*_glucose_: specific glucose uptake rate
S: syringic acid
SSF: simultaneous saccharification and ethanol fermentation
*q*_EtOH_: specific ethanol productivity
SEM: scanning electron microscopy
TEM: transmission electron microscopy
*μ*_max_: specific growth rate
V: vanillic acid
V + H: vanillic acid and *p*-hydroxybenzoic acid
V + S: vanillic acid and syringic acid
VHS: vanillic acid, *p*-hydroxybenzoic acid and syringic acid
*Y*_x/s_: biomass yield
*Y*_EtOH_: ethanol yield
