# Supplementary material for: Physiological mechanism of improved tolerance of Saccharomyces cerevisiae to lignin-derived phenolic acids in lignocellulosic ethanol fermentation by short-term adaptation
Source: Biotechnol Biofuels. 2019 Nov 14;12:268. doi: 10.1186/s13068-019-1610-9 (PMC6854637; doi:10.1186/s13068-019-1610-9)
Supplement: Supplementary file 1 — Additional file 1: Figure S1. Effect of phenolic acids on the specific growth rate. Figure S2. TEM imagines of S. cerevisiae after treatment of phenolic acids. [file 13068_2019_1610_MOESM1_ESM.docx]

**Additional file 1**

**Physiological mechanism of *Saccharomyces cerevisiae* improved tolerance to lignin-derived phenolic acids in lignocellulosic ethanol fermentation by short-term adaptation**

Hanqi Gu^1, 2, 4^*, Yuyong Zhu^1^, Yanfang Peng^1^, Xiaoguang Liu^1^, Lingzhi Shao^1^, Yanyan Xu^1^, Zhaohe Xu^1^, Ran Liu^1^, Jie Li^1^*

1. Department of Biology and Food Science, Hebei Normal University for Nationalities, Chengde, Hebei 067000, China

2. State Key Laboratory of Bioreactor Engineering, East China University of Science and Technology, 130 Meilong Road, Shanghai 200237, China

3. Basic Medical Institute, Chengde Medical University, Chengde, Hebei 067000, China

4. Department of Biological Systems Engineering, University of Wisconsin-Madison,

Madison, WI, 53706, USA

* Corresponding author:

Hanqi Gu, Tel/fax: +86-314-2370138, Email: [gu_hanqi@126.com](mailto:gu_hanqi@126.com)

Jie Li, Tel/fax: +86-314-2370080, Email: [lijie0651@163.com](mailto:lijie0651@163.com)

**Figure S1.** Effect of phenolic acids on the specific growth rate.

**Figure S2**. TEM imagines of *S. cerevisiae* after treatment of phenolic acids.


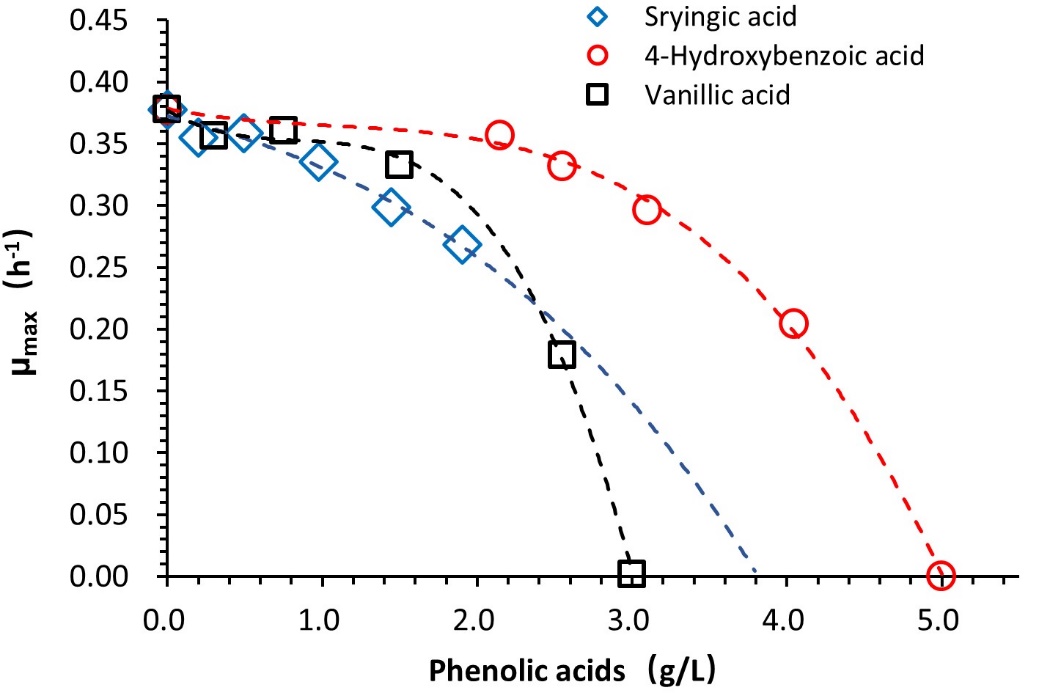


**Figure S1.** Effect of phenolic acids on the specific growth rate. The curves of specific growth rate against phenolic acid concentration was established based on the data of Table 1. The minimum inhibitory concentrations (MIC) was the lowest concentration of phenolic acid which could cause complete inhibition on yeast cell growth (specific growth rate was 0). For syringic acid, the concentration caused more than 30% of growth inhibition were not tested due to the low solubility, MIC was extrapolated through the fitting curve when the specific growth rate was 0 [1].


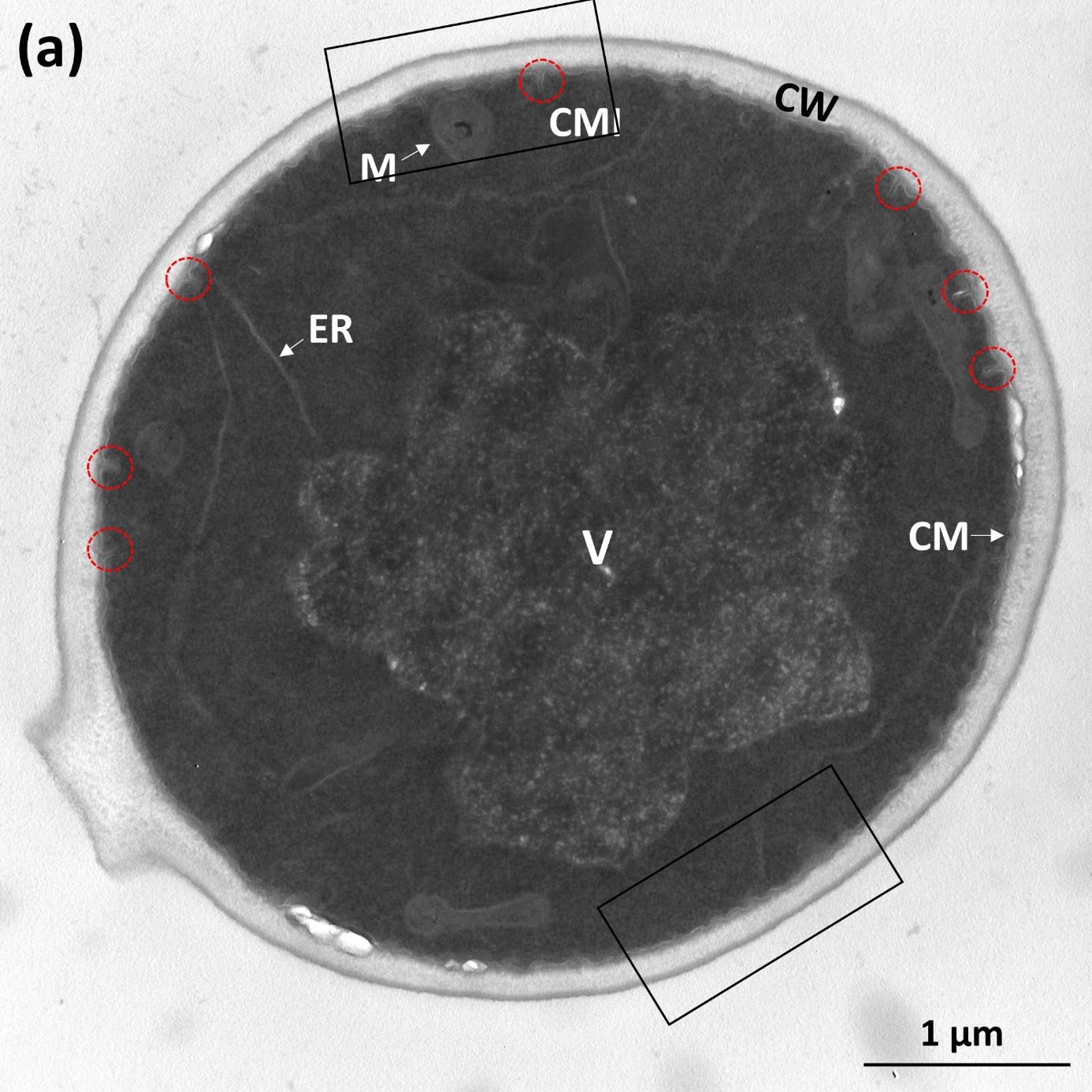


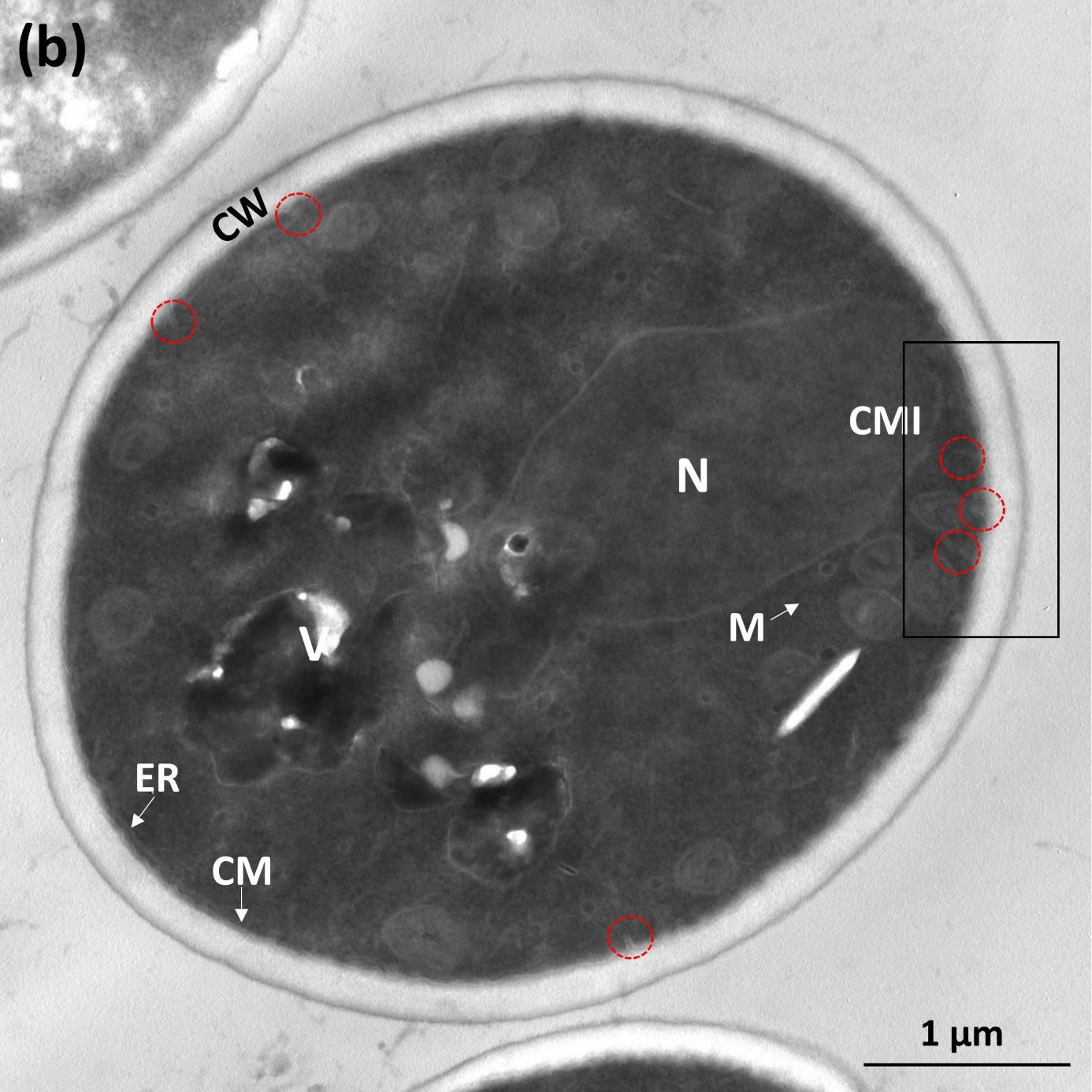


.


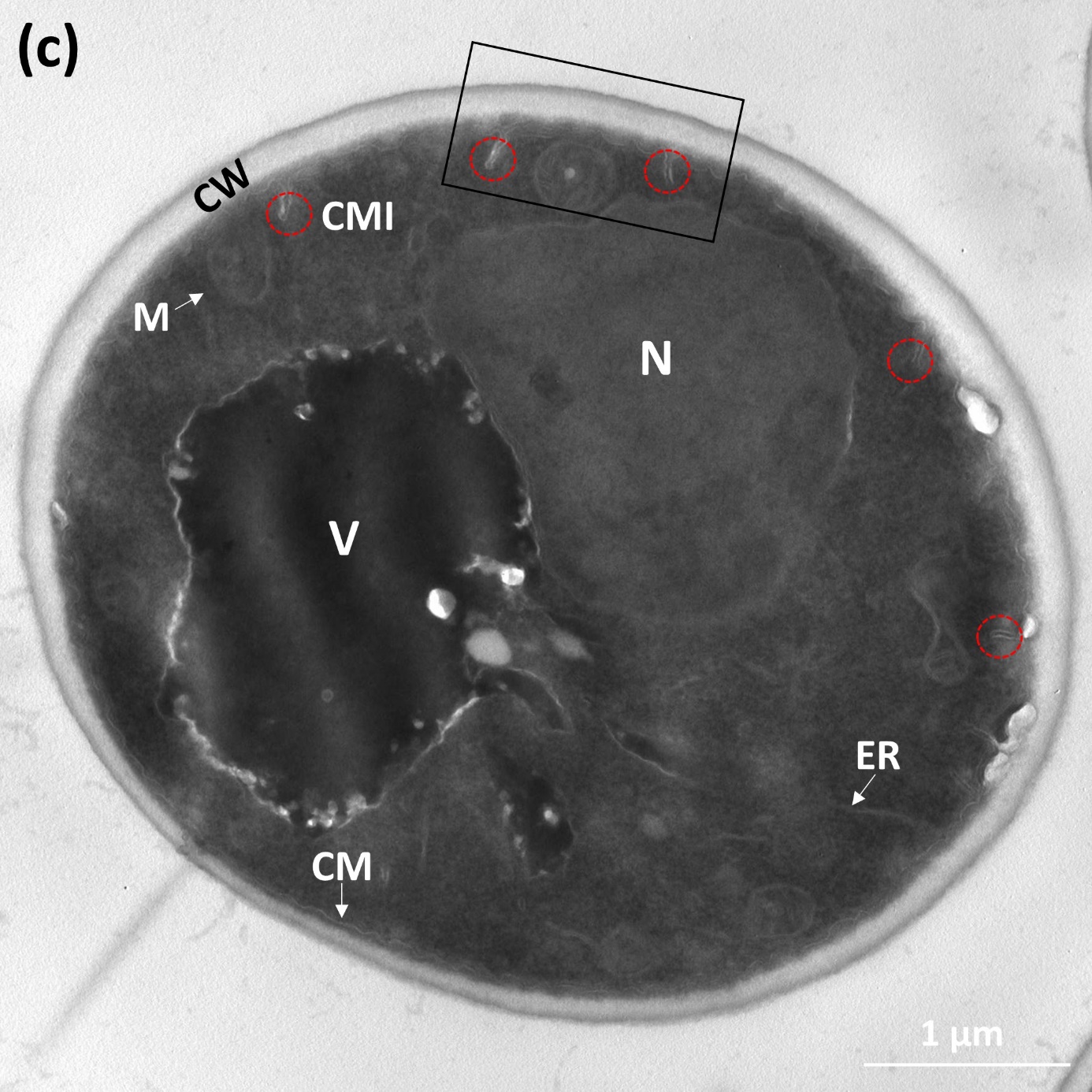


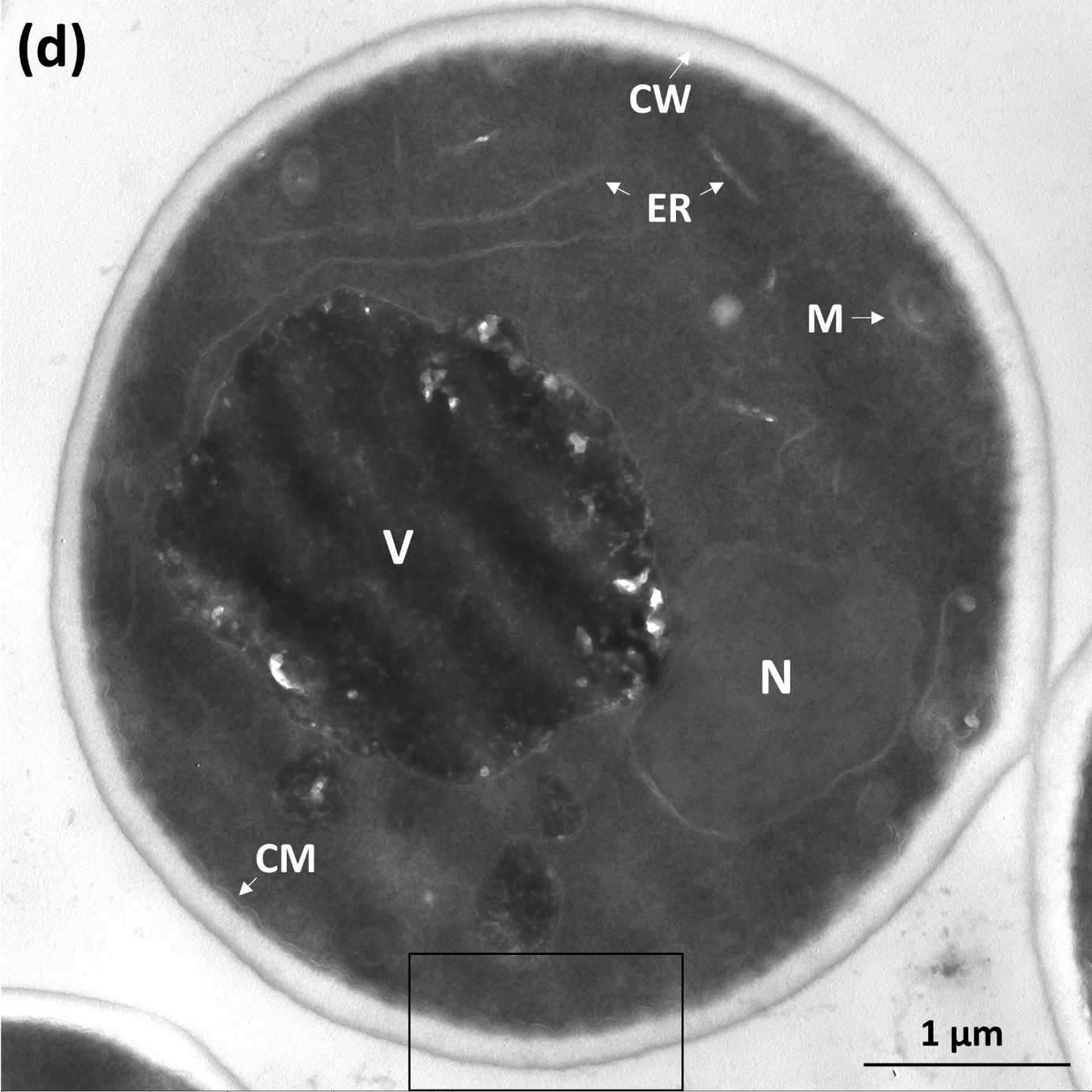


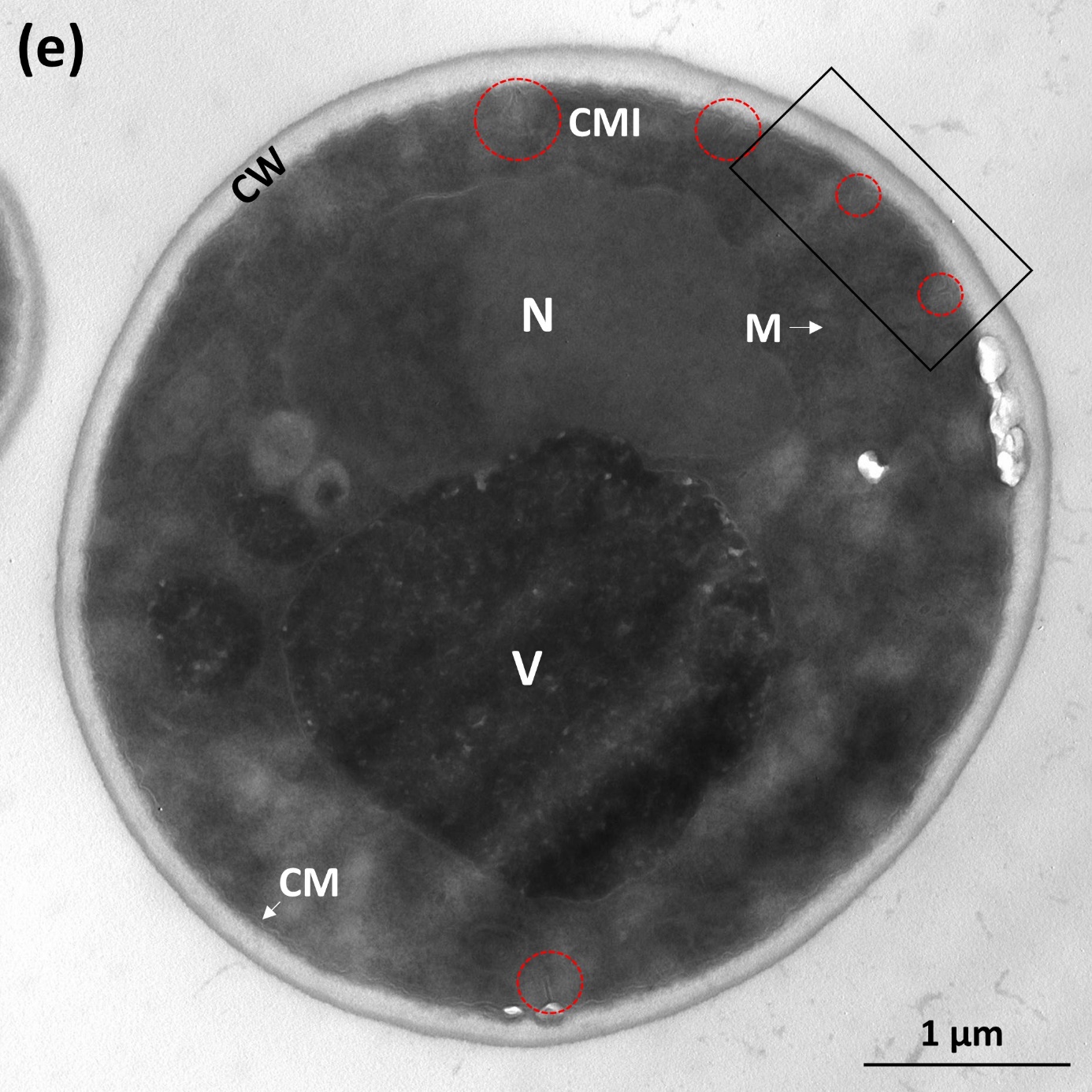


**Figure S2**. TEM imagines of *S. cerevisiae* after treatment of phenolic acids. (a) parental strain (PS) cells; (b) control group of adapted strain, which obtained from sequentially transferring and culturing yeast parental strain cells in synthetic medium without phenolic acid at the same condition with Method I; (c), (d), (e) were adapted strains from Method E, I and K, respectively. All above strain cells were inoculated in the medium with phenolic acids mixture at concentration of IC75, 30 °C, 150 rpm for 9 h. CW, cell wall; CM, cytoplasmic membrane; M, mitochondria; N, nucleus; V, vacuole; ER, endoplasmic reticulum; CMI, cytoplasmic membrane invagination.

**Reference**

1. Franden MA, Pilath H, Mohagheghi A, Pienkos P, Zhang M. Inhibition of growth of *Zymomonas mobilis* by model compounds found in lignocellulosic hydrolysates. Biotechnol Biofuels 2013, 6:99.
